# Conducting sexualities research: an outline of emergent issues and case studies from ten Wellcome-funded projects

**DOI:** 10.12688/wellcomeopenres.15283.1

**Published:** 2019-09-19

**Authors:** Dylan Kneale, Robert French, Helen Spandler, Ingrid Young, Carrie Purcell, Zoë Boden, Steven D. Brown, Dan Callwood, Sarah Carr, Alex Dymock, Rachael Eastham, Jacqui Gabb, Josie Henley, Charlotte Jones, Elizabeth McDermott, Nolwazi Mkhwanazi, James Ravenhill, Paula Reavey, Rachel Scott, Clarissa Smith, Matthew Smith, James Thomas, Karen Tingay

**Affiliations:** 1 EPPI-Centre, UCL Institute of Education, University College London, London, WC1H 0AL, UK; 2 School of Medicine, Cardiff University, Cardiff, Wales, CF14 4XN, UK; 3 Department of Infection & Immunity, Cardiff & Vale NHS Trust, Cardiff, Wales, CF14 4XN, UK; 4 Centre for Multilevel Modelling, University of Bristol, Bristol, BS8 1TX, UK; 5 School of Social Work, Care and Community, University of Central Lancashire, Preston, PR1 2HE, UK; 6 Centre for Biomedicine, Self and Society, Usher Institute, University of Edinburgh, Edinburgh, Scotland, EH8 9AG, UK; 7 MRC/CSO Social & Public Health Sciences Unit, University of Glasgow, Glasgow, G2 3AX, UK; 8 School of Applied Social Sciences, University of Brighton, Brighton, BN1 9PH, UK; 9 Faculty of Business & Law, Open University, Milton Keynes, MK7 6AA, UK; 10 University of Sunderland, Sunderland, SR1 3SD, UK; 11 Institute for Mental Health, University of Birmingham, UK, Birmingham, B15 2TT, UK; 12 School of Law, Royal Holloway, University of London, London, WC1B 3RF, UK; 13 Faculty of Health & Medicine, Lancaster University, Lancaster, LA1 4YW, UK; 14 Faculty of Arts and Social Sciences, Open University, UK, Milton Keynes, MK7 6AA, UK; 15 Wellcome Centre for Cultures and Environments of Health, University of Exter, Exeter, EX4 4PY, UK; 16 Department of Anthropology, University of the Witwatersrand, Johannesburg, 2000, South Africa; 17 Division of Psychology, London South Bank University, London, SE1 0AA, UK; 18 Department of Social Sciences, University of Sunderland, Sunderland, SR6 0DD, UK; 19 Centre for the Social History of Health and Healthcare, University of Strathclyde, Glasgow, G1 1XQ, UK; 20 UCL Institute of Education, University College London, London, WC1H 0AL, UK; 21 Office for National Statistics, Newport, NP10 8XG, UK

**Keywords:** sexualities, health, LGBT, Wellcome

## Abstract

This letter seeks to synthesise methodological challenges encountered in a cohort of Wellcome Trust-funded research projects focusing on sexualities and health. The ten Wellcome Trust projects span a diversity of gender and sexual orientations and identities, settings; institutional and non-institutional contexts, lifecourse stages, and explore a range of health-related interventions.  As researchers, we originate from a breadth of disciplinary traditions, use a variety of research methods and data sources. Despite this breadth, four common themes are found across the projects: (i) inclusivity, representations and representativeness, (ii) lumping together of diverse groups, (iii) institutions and closed settings (iv) ethical and governance barriers.

## Introduction

This letter seeks to synthesise methodological issues encountered in a cohort of Wellcome Trust-funded research projects focusing on sexualities and health. Further details of these projects are listed here: https://wellcome.ac.uk/news/10-new-projects-exploring-how-sexuality-affects-health.

The links between sexuality and health are complex and diverse ( Graugaard, 2017). Previous authors have explored future directions for sexuality research, highlighting challenges faced by those working in the field, such as a lack of suitable knowledge exchange and funding opportunities ( Irvine, 2014; Parker, 2009; Weis, 1998). Our concerns here are more applied, as we consider the methodological challenges encountered in undertaking research in this field.

The ten Wellcome Trust projects span a diversity of gender and sexual orientations and identities (cross-cutting binaries of male/female, gay/straight, masculine/feminine), settings (global south and north); institutional (from psychiatric wards to schools) and non-institutional, lifecourse stages (young through to older people), and explore a range of health-related interventions (abortion; mental health; and HIV pre-exposure prophylaxis (PrEP)). These challenges were identified during a Sexuality and Health meeting held during April 2018, at which all of the projects shared initial findings and discussed challenges faced in sexualities and health research.

As researchers, we originate from a breadth of disciplinary traditions (from anthropology through to biomedical studies), use a variety of research methods (including oral histories, ethnographic methods and meta-analyses) and data sources (from archival and secondary sources to the collection of primary data). Despite this breadth, common themes are found across the projects, and we group these under four headings:

- Inclusivity, representations and representativeness
- Lumping together of diverse groups
- Institutions and closed settings
- Ethical and governance barriers

The core of this letter is the sections about each of the four methodological challenges listed above. But before detailing these challenges, we give a brief overview of the context in which current sexualities and health research takes place. We follow this by drawing together the four themes, finishing with some ideas for future directions.

### Sexualities and health research

Human sexuality refers to the way in which people express themselves sexually and can include behavioural, social, relational, physical, erotic, biological, psychological, moral, and gendered dimensions of experience. While sexuality has played some part in all of our lives, the study of sexuality remains a relatively nascent field of research. From its origins, exploring public concerns around prostitution and venereal disease, sexuality researchers have increasingly critically interrogated sexuality ‘as a broad social domain involving multiple fields of power, diverse systems of knowledge, and sets of institutional and political discourses’ ( Irvine, 2014 p635).

While there is growing recognition around the complexity and diversity of sexual expression, researchers continue to work within climates that place boundaries on sexuality or overlook the influence of sexuality on many life course domains, including health. Narrow definitions of sexuality can reproduce prevailing power dynamics that have historically privileged sexual behaviour taking place within the context of monogamous, procreating sexual encounters, and stigmatising other sexual expression and behaviour as risky or dangerous ( Anderson, 2013), or overlooking these entirely. Critical sexualities research has challenged these conventions, leading to both an increased understanding and awareness of the diversity of behaviour and experience (i.e. not only procreative), as well as new understandings or re-conceptualisations of forms of sexuality, sexual expression and sexual relating (for example Dymock, 2012; Gabb & Fink, 2017; Parker, 2009; Ravenhill & de Visser, 2017). The links between sexuality and health are complex and diverse ( Graugaard, 2017). However, a great deal of health research relating to sexualities can focus on the negative consequences of sexuality, and it is important to also acknowledge positive aspects of sexuality. For example, there is some evidence that sexual satisfaction, sexual self-efficacy, sexual self-esteem and pleasure can enhance dimensions of, physical and mental health, and overall wellbeing ( Anderson, 2013).

The next section details the four methodological challenges we identified in the ten Wellcome-funded projects. We illustrate these challenges with excepts from each of the projects.

### Inclusivity and representations

All of the studies included within the Wellcome cohort are, in part, a response to the lack of representations of different sexualities, genders, social classes, ethnicities, geographies, and other intersections within the existing body of literature. Even where previous studies have purported to represent diverse groups, this has often resulted in the privileging or silencing of the experience of particular groups within that cohort. For example, although the health and care needs of older LGBT people as a whole are underrepresented within health literature; even within studies focussed on LGBT health, the experiences of bisexual and transgender people are often underrepresented. Similar issues of representativeness are recurrent across different intersectionalities, leading to under/over representation of groups within research literature.

All three excerpts from our case studies below outline how the issue of inclusivity brings specific challenges in ensuring representation of intersectional experiences of sexuality, across gender and gender identity (HS/SC), ethnicity (EM/JG/RE), and other issues of gender, sexual, socioeconomic and disability neutrality or normativity (IY).

**Case studies: the challenge of inclusivity within sexuality research**

In their historical project exploring the experience of lesbians, bisexual and non–gender conforming women in the mental health system ( Carr & Spandler, 2019), Helen Spandler and Sara Carr (HS/SC) describe the challenge of balancing the surfacing of certain experiences while avoiding silencing others. *‘One challenge we have faced is attempting to surface women’s experiences without reinforcing a fixed notion of gender or the prevailing gender binary. This means recognise and taking into account the conceptualisations and terminology used during the period in question. This is especially tricky with non-gender conforming women or potentially trans people. For example, should people who were assigned female at birth but live as men; and/or people assigned male at birth but lived as women, be considered part of hidden women’s history? There may be various ways to address this challenge, especially if we can find ways of more sensitively recording the complexity of gender identities and expressions. A queer theory inspired post-identity research strategy may seem to address this challenge, but still begs the question of how we find ways of recording specific groups of peoples experiences, so they are not lost or hidden.’*

The challenge described around developing an understanding of intersectional experiences of sexuality is one at the centre of Liz McDermott, Jacqui Gabb and Rachael Eastham’s (EM/JG/RE) research on how families and family relationships influence LGBTQ young people’s mental health and wellbeing. They describe how *‘Families come in many forms, and ethnicity, religion and socio-economic status are likely to be important factors in trying to unpack why and how family relationships are so significant to the mental health of LGBTQ youth. The problem with research in the UK is that much of the knowledge we have created on queer lives is based on overwhelmingly white, well educated (middle class) samples. Too often, researchers cite the mantra of the difficulties of recruiting a ‘hard-to-reach’ group, and this is used to justify a lack of attention paid to recruiting ethnically and culturally diverse samples. This matters because sexuality and gender diversity differ across cultures and ethnicities. There has been a tendency to homogenise LGBTQ experience as white. For example, many queer people of colour, Latino and indigenous people do not identify with terms LGBTQ. We live in a multicultural, globalised digitised world where people move across cities, countries and the globe (both physically and digitally) to find safer places to live and love. LGBTQ populations in any country are comprised of this queer migration. Our research samples should reflect this. In this study, we knew, through experience, that it was easier to recruit white participants than minority ethnic participants. We prioritised the recruitment of minority ethnic LGBTQ youth and designed a specific ethnic diversity recruitment strategy. This involved liaising with BAME youth workers, attending BAME youth groups and specifically addressing the ethical issues of confidentiality and anonymity which were particularly important to BAME LGBTQ youth.*’

Inclusivity is a challenge encountered in Ingrid Young’s (IY) research on sexual and biological citizenship. She describes how *‘sexualities research also struggles to engage with intersectionality adequately and to address how traditional patterns of social inequalities tend to map onto sexualities. There is a tendency to focus on women’s sexual health or gay men’s sexuality and not to consider how people may be affected by race, migration, mental health, other forms of illness, or socio-economic disparities. Quantitative surveys, where available, may go some way to identifying which variables (income, education, sexual identity, geographic location, gender) may be significant for sexual health outcomes, if and how these elements operate in combination with sexual practice and identity is less well understood.’*

### Lumping

‘Lumping’ refers to combining the experiences of diverse groups into a single category without accounting for, or overlooking diversity within the group. Despite its pervasiveness across projects, the underlying motivations for lumping differed. In quantitative research, lumping of people with diverse experiences into a monolithic category occurs because of a need to increase statistical power within analyses and to try to mitigate the possibility that differences in the data are obscured because of insufficient statistical power. In quantitative and qualitative research, lumping can also occur because of a need to simplify complex data through categorising behaviours, experiences and attitudes. These categories can make the analysis of complex research data easier to communicate, but often sacrifice attention to minority experiences. As the examples below also show, the values attached to these categories are often fixed and static, belying the complexity of the experiences of those to whom these categories are ascribed. As a result, while the motivations for ‘lumping’ are often intended to create more robust evidence, the practice can have the opposite effect. 

Although some degree of ‘lumping’ may be viewed as inevitable in any research, a heightened awareness of the consequences of this tendency may encourage future researchers to approach sexualities research in a more nuanced way. As we show below, lumping is a particular challenge in research exploring LGBT people’s experiences, which led to gaps in evidence and understanding around the health service experience and health needs of particular groups within the acronym, both historically (HS/SC) and present day (DK/RF). The practice of ‘lumping’ combines people with often very different needs and experiences, thus leading to research and practice which doesn’t address the needs of a such a diverse group as ‘LGBT people’. 

A present-day consequence of lumping meant that research has tended to focus on umbrella identities formed within a particular social, political and historical context, rather than experiences or behaviours (see IY, AD, and NM). This has implications for understanding the links between sexuality, health and wellbeing. As described clearly in one of the case studies below, the practice of ‘lumping’ in sexualities research has direct implications for the design of policy and programmes (NM), potentially leading to less effective policy and practice.

**Case studies: six examples exploring the impact of ‘lumping’ on sexuality research past and present**

Helen Spandler and Sara Carr’s (HS/SC) research on the experience of lesbians, bisexual and non–gender conforming women in the mental health system 1950s–1990s found that lumping *‘occurred on many different levels. For example, when we reviewed the medical, psychiatric and psychological literature on the subject, we found that sex or gender was rarely specified. [In addition] studies of ‘homosexuals’ and ‘transvestites’ rarely state whether they are referring to men or women. It seems it was generally assumed that both ‘homosexual’ and ‘transvestite’ referred to men, but this was rarely made explicit in the literature. The historic tendency to refer to people generically as ‘he’ means that women’s experiences may be lost. When women were included in studies their ‘data’ were frequently subsumed with the data on men and any unique differences, needs or issues are not highlighted or referred to. This makes it difficult for present-day researchers to be able to make gendered sense of the literature.’*

Dylan Kneale and Robert French (DK/RF) describe how a desire to avoid ‘lumping’ the experiences of a diverse group of people had stimulated the creation of their project exploring health and care inequalities among older LGBT people: *‘Previous quantitative research, including our own, has been compelled to ‘lump’ together lesbian, gay and bisexual older people within a single category because of limitations in sample size in datasets. By pooling information about older LGBT people across different datasets in a method known as Individual Participant Meta-Analysis, we are theoretically able to increase the statistical power and produce estimates with greater granularity across the LGBT acronym. However, in practice, because of the underrepresentation of older LGBT people in surveys, and because of historic conventions of omitting to ask older people about their sexuality, some lumping remains unavoidable. This means that we are still compelled to lump together lesbian/gay and bisexual women in a single category, for example, in many of our analyses where there may be strong grounds for assuming different health states and mechanisms. However, even distinguishing between men and women who are LGBT, is a substantial improvement on much previous research.’*

In Matt Smith and Dan Callwood’s (MS/DC) research on the impact of sexuality on mental health in sport, the breadth of the LGBT acronym also introduces substantial complexity: *‘the sheer diversity of experience within the LGBT community is both a challenge and an opportunity in researching and writing about LGBT sport and its effect on mental health – and this is true of work on sexuality as a whole. When taking a historical perspective, a particular problem is setting a balance between being inclusive and avoiding anachronism. For instance, even to talk of a coalition LGBT movement makes little sense before the 1990s, and strong tendencies towards separatism and antagonism between different sexual identities must be recognised rather than subsumed in a joint LGBT or queer identity.’*

In researching the links between sexual and biological citizenship, Ingrid Young (IY) found that *‘sexualities research about sexual health and HIV, tends to focus primarily on just a few, epidemiologically defined groups: gay and bisexual men, transgender men and women or heterosexual men and women. These ‘fixed’ categories, while usually (if not always accurately) indicative of ‘routes of disease transmission’, can mask socially and culturally specific sexual practices (where sexual acts have different meanings for different sexual partners) or fluid identities (where sexual behaviour is equated with more open identities). By going beyond these fixed categories and addressing the complexity of identities and practice within a health context, sexualities research can provide insight into how and why poor sexual health might be more than simply ‘people behaving badly’. Public health concerns with specific epidemiological groups and which focus on disease reduction and cost-effectiveness [which tend to involve lumping complex sexualities], are the main drivers of both funding and reporting demands and can significantly limit how sexualities, sexual health and wellbeing research questions are even formed, let alone considered.’*

Nolwazi Mkhwanazi’s project (WellSexualities), seeks to investigate the ways in which urban, black youth in South Africa experience and frame sexuality and what impact this has (or doesn’t have) on their use of sexual health interventions: *‘We particularly chose to work with urban, black youth because the majority of research, which is used as evidence in policy and programming in South Africa, tends to homogenize black, African youth sexuality by treating black South Africans as an undifferentiated whole. The research is often conducted among youth from resource-poor communities who are likely to be living in poverty. While the young people who participated in our study grew up in or lived in a township, unlike their parents, these young people all have tertiary education and could be described as middle-class. By specifically working with young people who live outside of the inner city, our project highlights the ways in which young people’s perceptions and experiences of sexuality change as they navigate the space of the township and the inner city; and the multiple performances of sexuality and sexual identities that are mobilized as they move between different spaces. Most importantly, our project underscores the fluidity of sexuality and cautions against static representations and understandings of sexuality within sexual health interventions.’*

Alex Dymock’s (AD) research challenges recent conventions of ‘lumping’ pharmacosexuality (sexuality and drugs) exclusively as an issue for men who have sex with men (MSM) in the literature. In her research, ‘lumping’ served to obfuscate the realities of lived experience: *‘The majority of research on drugs/sex repertoires concerns the sexual practices and communities of MSM. Our research aims to go ‘beyond’ this population by documenting the experiences of, and regulatory practices that have historically surrounded other populations. However, by not specifically focusing on different sexual identities, we risked obscuring some of the cultural and social specificities of these groups. However, as researchers particularly interested in ‘deviance’ and subcultures, we are keenly aware that focusing specifically on the behaviours of minority groups often has the undesirable effect of pathologising that group’s sexual practices and identities and promoting their over-policing and surveillance. Our recruitment strategy attempts to mitigate the problem of focusing only on a marginalised population.’*

### Closed settings and institutions

Individuals may spend a large amount of time within institutions, from schools and workplaces to the hospitals and care settings more common to health research. Within institutions, sexuality is often controlled, limited and stigmatised. Carers, medical practitioners, teachers, and other gatekeepers within institutions can problematise sexuality and serve to reinforce issues of underrepresentation discussed earlier. Furthermore, as demonstrated in one case study below (CS/RS), our attitudes and treatment of sexuality within institutions can reveal a great deal about our conceptualisations of sexuality in broader society; for example, a desire to control, as opposed to explore and promote sexual wellbeing. This is emphasised in a further case study (DK/RF), where social care settings are described as a nexus of sexuality-based health and care inequalities among older people. In addition, trying to find out what happened to LGBT people in institutions raises particular challenges because of historically changing policies and legal frameworks (HS/SC).

Institutional spaces pose particular issues for sexualities researchers, although access to these spaces is essential to ensure that research is representative of a diversity of experiences; and to ensure that policy-makers and practitioners have access to evidence that can help to promote the wellbeing, including sexual wellbeing, of people within institutions.

Despite the difficulties of conducting sexualities research in institutions, the case studies emphasise the need for sexualities researchers to continue to challenge institutional barriers and research sexuality in institutional spaces. As discussed below in the context of mental health settings (JR/PR), if researchers are not granted access to patients to discuss issues relating to their sexuality, policy development will be impeded and prohibitive practices to managing patient sexuality that are not conducive to positive mental health are likely to remain. Similarly, understanding the way in which sexuality was ‘treated’ in the recent past, can help to avoid the catastrophic errors in care that many have received within institutional settings (HS/SC).

As the excerpts below demonstrate, sexuality does not stop at the door of institutions and does not stop with the onset of mental or physical ill health or infirmity. The ‘closed door’ nature of institutional settings may become increasingly blurred through social media and increased digital intimacy. It is important for such changes to be managed in a way that accounts for the links between sexuality and wellbeing, and progresses beyond narrow controlling of self-expression and identity that has been associated with institutional environments.

**Case studies: undertaking sexualities research in institutional spaces**

James Ravenhill and Paula Reavy (JR/PR) describe significant barriers to accessing patients in their research exploring sexuality among people in secure mental healthcare settings, particularly those who have a background of criminal offending: *‘our capacity to conduct research has been obstructed by prevailing assumptions that discussions of sexuality and intimacy are destabilising for people in secure settings. It is assumed that research might disturb the “institutional discipline” of the wards, is inappropriate, or perhaps of greater concern, irrelevant. Evidence from existing research suggests that successful intimate relationships can play an important role in promoting positive mental health and recovery from mental distress. But our research has identified that patients’ understandings of how their subjective, “felt” experiences of sexuality are related to their mental health and their recovery is inhibited by a culture of silence surrounding their need for (and right to) sexual expression. This situation is exacerbated by providers’ disinclination to allow researchers to talk to their patients about these issues. For people who experience mental distress, but who also have a background of criminal offending, discourses of risk, vulnerability and predation arise that serve to shroud discussions of sexuality in even greater uncertainty. Attempts at providing personalised mental healthcare that accounts for patient sexuality are therefore obstructed, and the sexual expression of forensic patients in secure settings is often “driven underground”. The findings from this study will contribute to a programme of research aimed at informing the development of national policies to guide the management of relationships and sexuality in secure mental healthcare.*

Clarissa Smith and Rachel Scott’s (CS/RS) research takes place within a changing context around how sexuality will be treated within schools: *‘Discussions of young people and their consumption of sexual media remain fraught, particularly in policy arenas, but school-based sex and relationships education will become compulsory in England in 2019 and will include topics like pornography, sexting and use of online media. How those will be taught is not clear, nor is there sustained research evidence available for best practice. Too often research is predicated on fears, for example of the effects of pornography on young people and solutions proposed which centre on the best ways to curtail young people’s explorations. Our approach has been to seriously examine the changes technologies have wrought on the ways young people communicate and connect, as well as how those technologies have offered new opportunities for intimate practices. These digital intimacies encompass a wide range of practices, including producing, sharing, broadcasting and viewing intimate content such as sexting; taking and sharing selfies; using hook-up apps; communicating about sex and relationships; searching for information and advice; and creating, accessing and circulating sexual content online, through social media and through apps.’*

Helen Spandler and Sara Carr (HS/SC) have encountered a number of research challenges in trying to establish what happened to lesbians, bisexual and non–gender conforming women in the mental health system in the UK: *‘We are especially focusing on the post-war period when psychiatric and psychological interventions were used to treat ‘sexual deviation’. We know very little about what happened to women during this time due to the different legal context of male and female homosexuality. Male homosexuality was criminalised and men who were arrested for homosexual activity could be offered NHS psychiatric ‘treatment’ instead of imprisonment. Therefore, documentation in the form of court orders and court referrals exist which can be used to establish the extent and range of treatments men received. Women’s same-sex relationships were not criminalised therefore these pathways to treatment did not exist. This does not mean women didn’t receive these treatments - we know that they did, but in smaller numbers, at least partly because of this legal context. This makes it harder to find examples in any records. In addition, sexuality and gender orientation were rarely recorded in hospital records. This makes is hard to trace back from psychiatric patient records or accounts. From the information we have gathered it isn’t always easy to establish if the psychiatric treatment a person received was given to treat their sexuality or if it was for a condition or behaviour only indirectly related, or even unrelated. Establishing links between mental distress, treatment and sexuality in written records would have been difficult enough to discern at the time, but is even more of a challenge to determine in retrospect. It is also difficult to establish whether any ‘treatments’ received were voluntary, coerced or forced. Moreover, given the background of the historical sexual oppression and control of women, the border between consent and coercion is not easy to establish. Finally, if psychiatric treatment were ever ‘successful’ (sic) in orientating people to a heterosexual or gender-conforming lifestyle, they would be subsequently hard to identify though LGBTQI archives, if at all.’*

Dylan Kneale and Robert French (DK/RF) are exploring the impact of sexuality on health and care inequalities in later life: *‘as a first step in our research, we conducted a systematic scoping review exploring where other researchers had identified health and care inequalities among older LGBT people. Our review included studies that had adopted a wide variety of different research methods. The evidence suggested that social care environments were a nexus for the emergence of health and care inequalities. For many reasons, older LGBT people may be more likely to enter formal care environments, and having entered these environments, to experience adverse care outcomes because of heteronormativity and homophobia. However, our quantitative study is not able to examine these findings further because of the absence of data about older people in care environments. An absence of data collected from social care settings is not an issue that is only specific to LGBT people. However, because of the potentially heightened risk of older LGBT people entering these environments, and because of the inequity in treatment many face having entered institutional spaces, the absence of these data is particularly harmful to our aim of understanding older LGBT people’s health and care needs. Our study is nevertheless helping to understand the magnitude of other health and care inequalities such as inequalities in experience of illness and (un)healthy lifestyles.’*

### Ethics and governance issues

Sexualities research raises additional issues in research governance; for example, in the granting of ethical approval and collecting or obtaining data. 

These challenges can begin even within our University institutions, where research exploring sexual practice and sexual identity can be viewed as ‘sensitive’ and subject to additional scrutiny (e.g. CS/RS) and greater legal restrictions on how such data is held, processed and shared. While we would not seek to downplay the importance of conducting ethically sound research, some of the case studies below contrast the perceptions of ethics committees with those of research participants (e.g. CP). In addition, blanket rules around the publication of quantitative data enforced by ethical committees and other bodies, such as suppressing small numbers to preserve potential breaches in confidentiality, while ethically sound, can inadvertently compound the issues around inclusivity and ‘lumping’ described above, leading to poorer quality evidence available for decision-makers (DK/RF).

The governance arrangements set in place around working with administrative data in sexuality and health research are particularly stringent. The legal restrictions are complex in England and Wales (similar arrangements exist in Scotland and Northern Ireland). There are two legal frameworks (i) the common law duty of confidence and (ii) data protection law (GDPR & Data Protection Act), both of which must be satisfied for processing of personal data for health and social care research. Under the common law duty of confidence, information about sexual orientation is considered ‘confidential information’. To process confidential information, researchers have to rely on consent for disclosure in line with common law, or seek Section 251 support from the Confidentially Advisory Group (CAG) at the Health Research Authority (HRA) to set aside the common law duty of confidentiality. Under data protection law, sexual orientation would be considered not just personal data, which requires a legal basis for processing (typically ‘public interest’ for university research or ‘legitimate interests’ for commercial or charity-based research), but ‘special category’ personal data. This adds three further conditions for processing including compliance in terms of: (i) ‘necessary purposes’; (ii) ‘subject to appropriate safeguards’; and (iii) ‘in the public interest’. The legal difficulties in processing personal data mean large publicly funded institutions which act as repositories for linked large administrative datasets are unable to retain sexuality in their datasets. The Secure Anonymised Information Linkage centre, funded in part by HDR-UK, the most advanced national data linkage repository in the UK, has excluded any recording of sexuality across all datasets (in addition to sexually transmitted infections).

In some cases, assumptions about the inherent sensitivity of data on sexuality made by data controllers can lead to inconsistency in the types of data made available to researchers. In turn these inconsistencies in data access can again compound issues of representation and inclusivity, or can lead to methodological issues around statistical power in analyses of minority populations (DK/RF, AD).

**Case studies: research ethics and governance challenges faced in sexualities research**

Carrie Purcell’s (CP) research on abortion touches on several dimensions of governance and ethical issues: *‘a key issue with researching abortion - and potentially other sexuality-related phenomena - is that it is presupposed to be a ‘sensitive’ and ‘controversial’ issue. The issue of ‘sensitivity’ can have implications for obtaining ethical approval for primary studies, where ethics committees have concerns regarding, for example, whether asking women about their abortion experiences might cause undue harm, or whether it is appropriate to ask younger people their views on the subject at all. While it is, of course, essential that sexualities (indeed any) research approaches participants in a considered and respectful way, foregrounding ‘sensitivity’ above all else implies that abortion is somehow so dangerous that simply talking to a researcher about it may be damaging. This perspective arguably contributes to the silences around abortion which perpetuate stigma. The related issue of ‘controversy’ has similar implications. This tends to be the default framing of abortion in the news media, as has been seen frequently in the UK and Ireland. However, this conflicts with the fact that abortion is a commonly conducted gynaecological procedure, and has been a part of routine women’s healthcare for over half a century. For many of the women who undergo it, it would not be ‘controversial’, but the prevailing narrative frames it this way. Amongst other things, our current project explores how such negative orientations to abortion can be resisted and rejected, and how more positive framings might be normalised, replacing the current default view’. *

Alex Dymock’s (AD) research exploring pharmacosexuality (sexuality and drugs) through digital ethnographic methods reports that *‘an ongoing concern raised about the ethics of ‘lurking’ as a research method. Our project involves using ‘digital trace’ data from drugs forums. There is currently a debate in the literature, specifically related to drugs research, about whether participants in such spaces, and the data they produce, should be considered as they would in any other ethnographic research, and therefore that identities should be fully protected; or whether they should be considered identifiable actors, with digital property rights over the data produced in such spaces. Our ethics application is currently under review, but we are still considering our approach.’*

Clarissa Smith and Rachel Scott’s (CS/RS) research on young people and their consumption of sexual media has involved *‘examining the evidence base on young people and digital intimacies and collaborating with key people from research, policy and practitioner backgrounds in order to advance the future research agenda on young people, digital intimacies and sex education. Since much of current research and practice focuses on the risks and harms of technology and sexual life it is important that we interrogate assumptions of risks and harm, and recognise that these narratives can in themselves be harmful. Building research and practice around an ethical framework may be a productive way of critiquing the harm narrative and an effective means of redirecting the conversation away from risk and harm and towards providing supportive spaces and interventions for young people. One means of doing that is to design inclusive research which involves young people, and the professionals who work closely with them, right from the start – too often research into young people’s practices starts from the idea that ‘something must be done’ and does not address the particularities of young people’s interests in, and experiences of, intimacies, sex and sexuality whether digital or offline. Instead of talking about how to protect young people, the focus could shift to what would ‘good’ sex, friendships relationships or intimacies might look like for a young person, and what is needed to support this.*

Dylan Kneale and Robert French (DK/RF) are marshalling diverse data sources to understand sexuality-based health and care inequalities in later life. They describe how different data depositors appear to have wildly different policies around classifying sexuality data as ‘sensitive’ which impacts on its availability. *‘For example, a data collector of one long-standing series of surveys started collecting data about sexuality in 2010, although after 2014 this data was no longer being made available to researchers through any form of secure license, despite being collected. The decision to stop access to the data can be perceived as unethical from two different standpoints. Firstly, it can be viewed as unethical to collect data from participants without making it clear that this data will not be used. Secondly, it can be viewed as unethical to store data about participants’ sexuality out of reach of trained data users, where it could be used to inform decision-making and improve services from the very people from whom it has been collected. Adults offering information about their sexuality in surveys do so knowingly and with full consent; it is not clear why others involved in later stages of research governance view these data about sexuality as highly sensitive and banish it out of reach from researchers. It also begs the question of why data collected about sexuality in 2014 is not sensitive, but the same data collected a year later, albeit from a different sample, is considered sensitive.’*

## Conclusions

The experience of Wellcome-funded researchers, upon which this letter is based, raises four key challenges for sexuality research:

1. Ensuring inclusivity in the representation and representativeness of research participants, especially around historically underrepresented categories and intersectionality.
2. Avoiding ‘lumping’ diverse experiences and identity under a single category or construct, (e.g. LGBT) with little attention to the important experiential variations within this category.
3. Developing a better understanding of the relationship between sexualities and health within institutional settings where sexuality is regulated and controlled
4. How to appropriately negotiate, or challenge research governance and ethics procedures which can serve to stigmatise common forms of sexuality, sexual behaviour and sexual expression.

Sexualities research implicitly involves asking challenging questions. Rather than shying away from historical, personal and political complexity, researchers can emphasise the experience of sexual variation in society, and explore how that relates to health. Approaching sexualities research with an expansive lens and an eye on historical context also helps researchers to avoid the stigmatisation that unwanted categorisation might bring. Nevertheless, exploring these questions has historically been regarded as an esoteric pursuit, despite the substantial implications for population health.

### Future directions

The Wellcome-funded research projects cited in this letter have the potential to contribute to the development of national policies. We hope that research councils and other funders closely monitor the results and impact of these pilot projects and consider similar initiatives that allow researchers to explore relationships between sexualities and health, across different settings and contexts and which take into account underrepresented experiences and/or identities.

To facilitate future research, we make the following suggestions for improving sexualities research in the future:

1. Sexualities research implicitly involves working with diverse groups with complex behaviours and experiences. While categorising these or giving them acronyms can be a useful starting point in attempting to make sense of complexity, overreliance on these masks heterogeneity within groups, and attaches static values to these identities. As researchers, we need to find ways of honouring self-identities, experiences, and desires.
2. To ensure inclusivity within future LGBT research, Based on their research, Liz McDermott, Jacqui Gabb and Rachael Eastham emphasise the importance of: (i) developing a commitment to an ethnically diverse sample; (ii) finding out and understanding what is necessary to be successful; (iii) manage expectations/realistic goals as it is resource intensive; (iv) get advice from those who know, e.g. BAME youth workers.; (v) recruit ethnically diverse researchers; (vi) monitor sample, if you fail, reconfigure your strategy, e.g. stop recruiting white participants.
3. Sexualities research is often hampered by a perception that it involves ‘risky’ or ‘sensitive’ subjects, even though research participants do not always share this perception. This perception can impede funding opportunities and applications, ethics approval, and obtaining and collecting data. Ethical issues in sexualities research could be better considered by researchers and lay members with expertise in sexualities research, who currently may not feature on many institutions’ ethical approval boards. We would also encourage those appraising sexualities research to look beyond the ‘sexuality’ aspect of the research and to consider the research merits of individual projects.
4. Sexualities researchers need to be mindful of linguistic and other traps which perpetuate stigma and the marginalisation of certain aspects of sexual expression. We also need to develop a better understanding of what works in informing and changing policy and practice. This could include developing case studies which have developed ways of communicating research findings which do not reinforce negative tropes and stereotypes about sex and sexuality.
5. We need to consider the impact of the legal restrictions on processing sexuality data for research. Policies around access to data about sexuality should be based on clearer assessment criteria, rather than the ad hoc criteria that appears to be imposed on many datasets currently. It is important to find the balance between protecting privacy with the potential benefits of research.
6. All researchers need to be mindful of future-proofing. Helen Spandler and Sarah Carr’s research reminds us that we need to ask ourselves questions such as: what are the current ways that researchers collect and archive data that might make it difficult for future researchers? While it is impossible to completely ‘future proof’ research – we do not know what social changes lie ahead – we can certainly be more sensitive to the challenges highlighted here and ensure that we carefully record important characteristics for future generations of researchers. They are attempting to resolve challenges in in their research by taking their cue from feminist organisations and researchers who simultaneously seek to draw attention to the specificities of women’s experiences while also being trans-inclusive. As researchers, we do not know how people may seek to express, define and understand their gender and sexuality in the future. However, we do know that it is very likely to shift and change in ways that we might not anticipate.

There are no easy solutions to these challenges, but by raising these issues, we hope to draw attention to the opportunities and barriers to developing new forms of knowledge about sexuality and provoke debate and discussion about how best to achieve this. Our collective work underscores the fluidity of sexuality and cautions against static representations and understandings of sexuality within sexual health interventions and research. Recognising this fluidity will improve our understanding of sexuality, and further knowledge about the links between sexuality, health and wellbeing.

## Data availability

No data are associated with this article.
