# Supplementary material for: Conducting sexualities research: an outline of emergent issues and case studies from ten Wellcome-funded projects
Source: Wellcome Open Res. 2019 Sep 19;4:137. [Version 1] doi: 10.12688/wellcomeopenres.15283.1 (PMC6961412; doi:10.12688/wellcomeopenres.15283.1)
Supplement: Peer review history

# Peer review: Reviewer response for version 1

**DOI:** 10.21956/wellcomeopenres.16681.r37021

**Published:** 2020-01-13

**Authors:** Robert Pralat

**Affiliations:** 1 Department of Sociology, University of Cambridge, Cambridge, UK

This open letter provides an overview of “methodological challenges” as identified by researchers from ten projects funded by the Wellcome Trust and focused on health and sexuality. The challenges are grouped under four themes (“inclusivity, representation and representativeness”; “lumping together of diverse groups”; “institutions and closed settings”; “ethical and governance barriers”), each illustrated with case studies from the ten projects. The projects span different disciplinary and methodological approaches (including various social and health sciences, as well as qualitative and quantitative methods) and cover a range of topics (including abortion, drug use, mental health and ageing, to name a few). The letter concludes with six “suggestions for improving sexualities research in the future”.

 The letter is a useful, practically focused and accessibly written intervention into a growing area of research. What makes the letter compelling is its format as it allows the authors to bring together a variety of perspectives from scholars who are at the cutting edge of sexuality research, each working on a study with important implications for health and wellbeing. The letter effectively conveys both the significance of this work and the difficulty of doing it. The authors are honest about the various dilemmas and complexities they have faced in their projects, showing that, while there may rarely be “right” answers to methodological conundrums, there are nevertheless ways of improving the overall quality of work on sexuality in health research. Paying closer attention to the issues the letter identifies is a good way for researchers to start.

 I enjoyed reading the letter and I have no major criticisms. My main suggestion would be to clarify the audience. Who exactly is the letter written for? Researchers, including graduate students, are the obvious audience, but this could still be specified at the outset. Towards the end of the letter, the authors mention research councils and other funders, and this audience could be highlighted earlier on too. Ethics board members might be another part of the readership worth mentioning. I think the importance and usefulness of the letter could be better conveyed in the abstract and the first paragraph of the introduction. Currently, the opening paragraph directs the reader’s attention elsewhere (a website with further details about specific projects) and it could be more engaging (for example, by giving a brief overview of the projects’ thematic scope). After all, Wellcome had identified “sexuality” as a priority research area, so it would be good to start (and finish) the letter on a stronger note to make the significance of this work more explicit. The letter would also benefit from closer proofreading as it currently contains a number of typos and grammatical/punctuation errors (especially in the case study boxes).

 Overall, I very much welcome this open letter and the opportunity to comment on it. The authors should be commended for using this format to share the various insights from their work and consolidate them into succinct themes, which will hopefully guide current and future researchers studying health and sexuality.

I confirm that I have read this submission and believe that I have an appropriate level of expertise to confirm that it is of an acceptable scientific standard.

# Peer review: Reviewer response for version 1

**DOI:** 10.21956/wellcomeopenres.16681.r36527

**Published:** 2019-12-18

**Authors:** Ralf Lottmann

**Affiliations:** 1 Alice Salomon University , Berlin, Germany

This open letter uses ten Wellcome Trust funded research projects to identify challenges and important aspects of research on sexuality and health. It is well written and easy to read.

 This letter provides a collection of learning effects through a diverse range of research projects with different topics and methodological research designs. It has used an interesting idea to find similarities and differences among those projects in order to gain knowledge that can be used for future research on sexuality and health. Researchers can also learn from those projects to avoid mistakes, to improve research on LGBT issues and to handle key challenges for sexuality research carefully.

 The diverse research projects provide a solid basis for an extensive presentation of findings under four headings that are plausible and informative (Inclusivity, representations; Lumping together of diverse groups; Institutions and settings; Ethical and governance and barriers). This amount of portrays of projects limits the space that could have been used for some additional information which might be useful to understand challenges and future directions a little bit better. In the following, I will mention a few aspects that could be added to three of the four headings.

 Under inclusivity, the researchers present interesting case studies that are useful to describe challenges in ensuring representation of intersectional experiences of sexuality. The case studies might be more understandable if at least one example could be presented. We learn that there was a specific ethnic recruitment strategy used (case study 2) but more information about the research design is missing. In order to avoid mistakes and to improve future research on that topic, the research design (or their interventions) could be such an example.

 Lumping: It is an interesting chapter about problems due to combining diverse groups that can lead to a lack of diversity and representation. I wonder if qualitative research projects "simplify complex data" through categorising behaviour, experiences and attitudes the same way as quantitative research projects. It would be useful if the researchers could differentiate between those methodological schools or just mention the examples/case studies of social research in general. And even if "fixed categories" (see case study IY) are used findings more than "simply people behaving badly" are possible.

 Closed settings and institutions:

 This is a very interesting chapter about practical relevance of research on sexuality and health. Instead of "sexuality does not stop at the door of institutions" it might be better to use a wider term such as sexual and gender identity.

 Conclusions: To summarise the conclusions and suggestions for future research is an important and very useful part of the letter. I do not know the intention of an open letter very well but it might be interesting if some references could help to connect and to integrate those interesting findings/suggestions to the existing literature.

I confirm that I have read this submission and believe that I have an appropriate level of expertise to confirm that it is of an acceptable scientific standard.

# Peer review: Reviewer response for version 1

**DOI:** 10.21956/wellcomeopenres.16681.r36659

**Published:** 2019-10-22

**Authors:** David A. Griffiths

**Affiliations:** 1 Department of Sociology, University Of Surrey, Guildford, UK

This letter provides insight into ten Wellcome Trust funded projects on sexualities and health. The letter is accessible, well-written and easy to read. This letter will be of interest to anyone involved in sexualities studies, and I think it is a particularly useful resource for those about to start research projects who are reflecting on methodologies and ethics.

 The letter considers methodological problems encountered in a range of different projects focused on sexualities and health. The projects focus on a fascinating diversity of topics and contexts, from a broad range of disciplinary backgrounds and considers sexuality and health in interesting and expansive ways.

 Despite this, the authors provide insight into methodological challenges that occur across the projects, and do a very good job of synthesising these into four clear themes.

 In the first section, ‘Inclusivity, representations and representativeness’ the authors emphasise that all the projects discussed are a response to a lack of representation of some sort, but that they each faced methodological issues around inclusivity and representativeness. The second section, ‘Lumping’, develops this theme, arguing that combining diverse groups into meaningful categories for research is unavoidable, but must be reflected upon particularly in light of issues of diversity and representation. The third section, ‘Closed settings and institutions’, considers institutions from schools to hospitals and the particular challenges associated with research in these contexts, but also the great potential in this research. The final theme is ‘Ethics and governance issues’. This is a very important issue for sexualities studies. The authors do an excellent job of carefully negotiating the importance of ethical research, while highlighting the fact that ethics boards and participants may have very different views about what constitutes good and useful research, and that ethically sound rules and procedures can sometimes negatively affect the methodological challenges previously outlined. This is nicely illustrated by the case study research on abortion, where the very act of defining the topic as “sensitive” can have a stigmatising and othering effect. This is an unresolved issue in sexualities studies, and is an important insight of the letter.

 The inclusion of key (and honest) insights from the research projects as case studies is a particular strength of this letter and illustrates clearly the dilemmas facing researchers in this field. While the letter synthesises these challenges clearly, it also avoids making prescriptive judgements about particular methodologies or approaches. Indeed, it is interesting and useful to see the different individual responses to different issues, whether through methodologies, recruitment strategies or other approaches.

 The authors conclude with six suggestions for future research directions. These are clearly stated, and I hope that researchers, funders and ethics boards take note!

 Overall, this is an interesting insight into cutting-edge research into sexualities and health that offers useful reflection on methodological challenges and clear directions for future research.

I confirm that I have read this submission and believe that I have an appropriate level of expertise to confirm that it is of an acceptable scientific standard.
